# Supplementary material for: Gene Expression Profiling of Fibroepithelial Lesions of the Breast
Source: Int J Mol Sci. 2023 May 20;24(10):9041. doi: 10.3390/ijms24109041 (PMC10219050; doi:10.3390/ijms24109041)
Supplement: Supplementary file 1 [file ijms-24-09041-s001.zip › ijms-2399307-supplementary.pptx]

## Slide 1
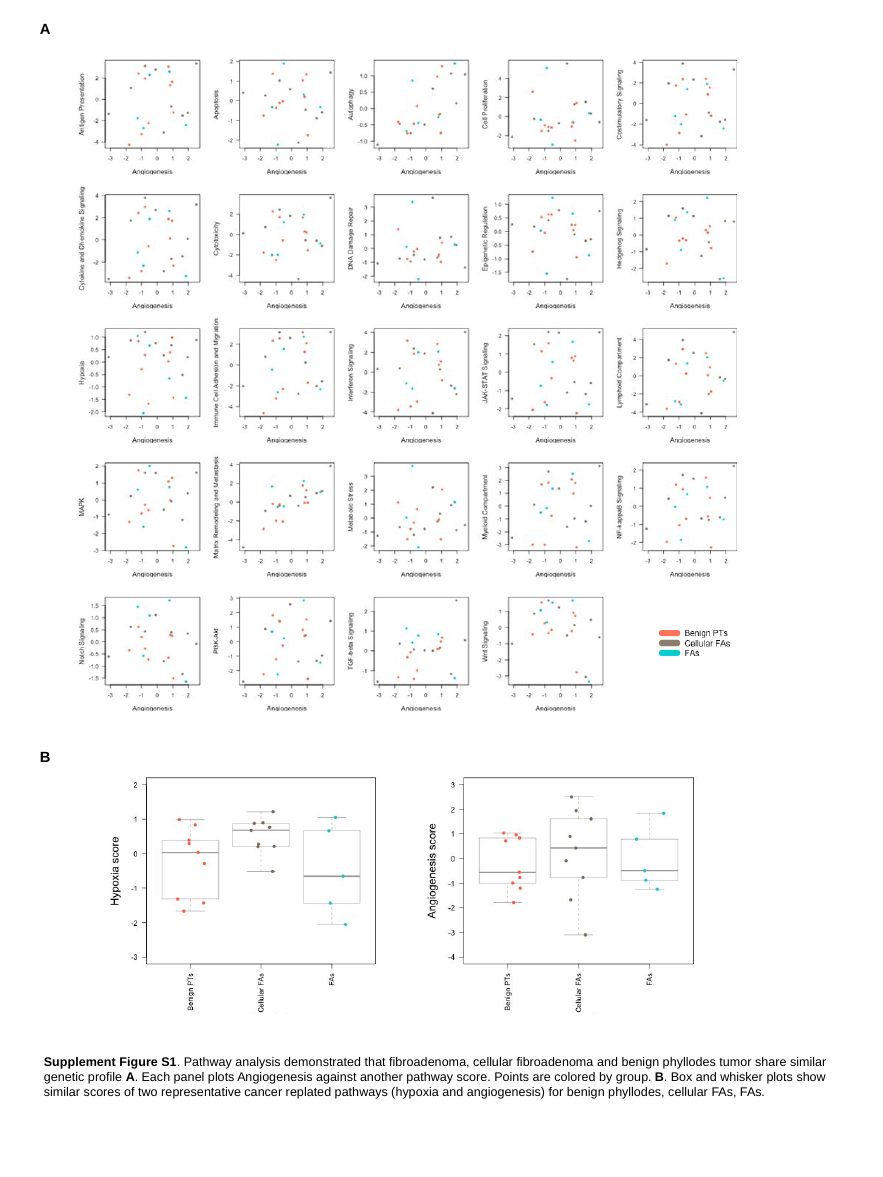

A
B
Supplement Figure S1. Pathway analysis demonstrated that fibroadenoma, cellular fibroadenoma and benign phyllodes tumor share similar genetic profile A. Each panel plots Angiogenesis against another pathway score. Points are colored by group. B. Box and whisker plots show similar scores of two representative cancer replated pathways (hypoxia and angiogenesis) for benign phyllodes, cellular FAs, FAs.

## Slide 2
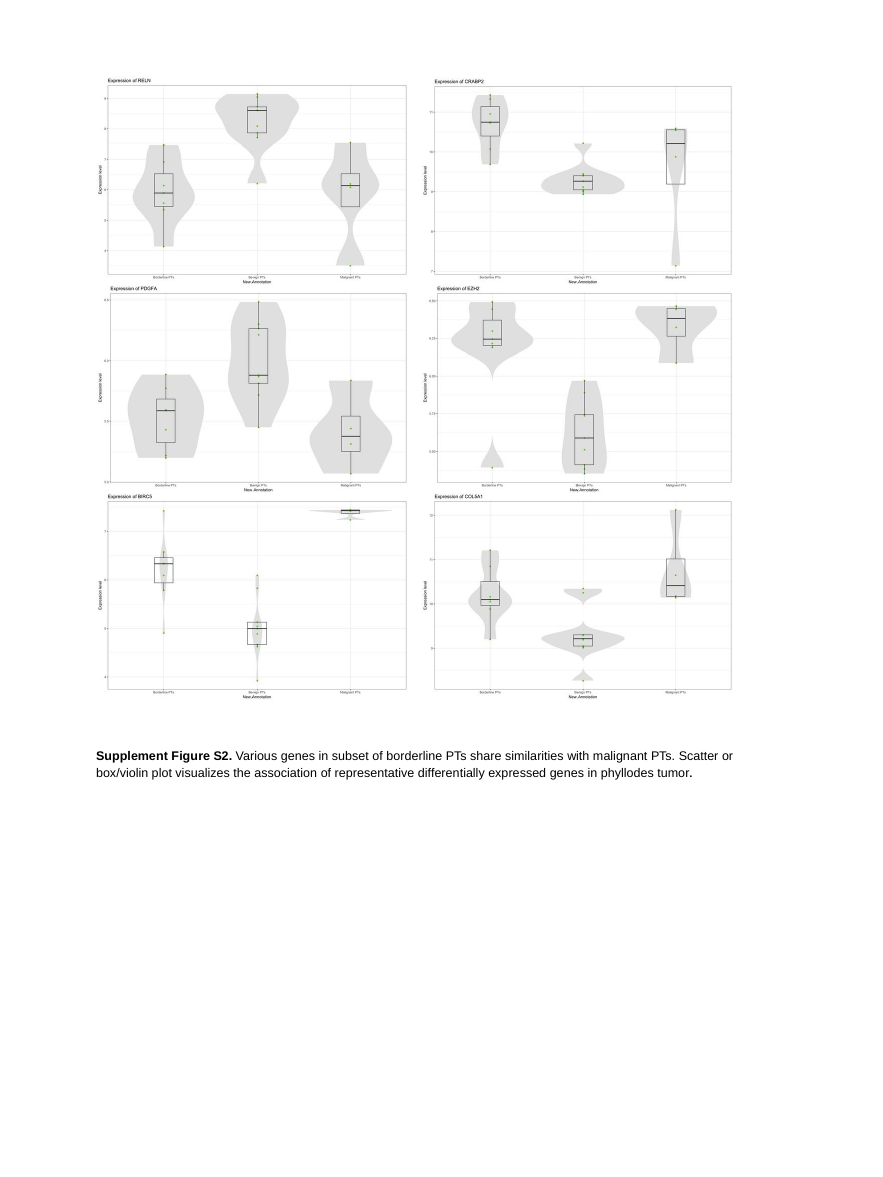

Supplement Figure S2. Various genes in subset of borderline PTs share similarities with malignant PTs. Scatter or box/violin plot visualizes the association of representative differentially expressed genes in phyllodes tumor.

## Slide 3
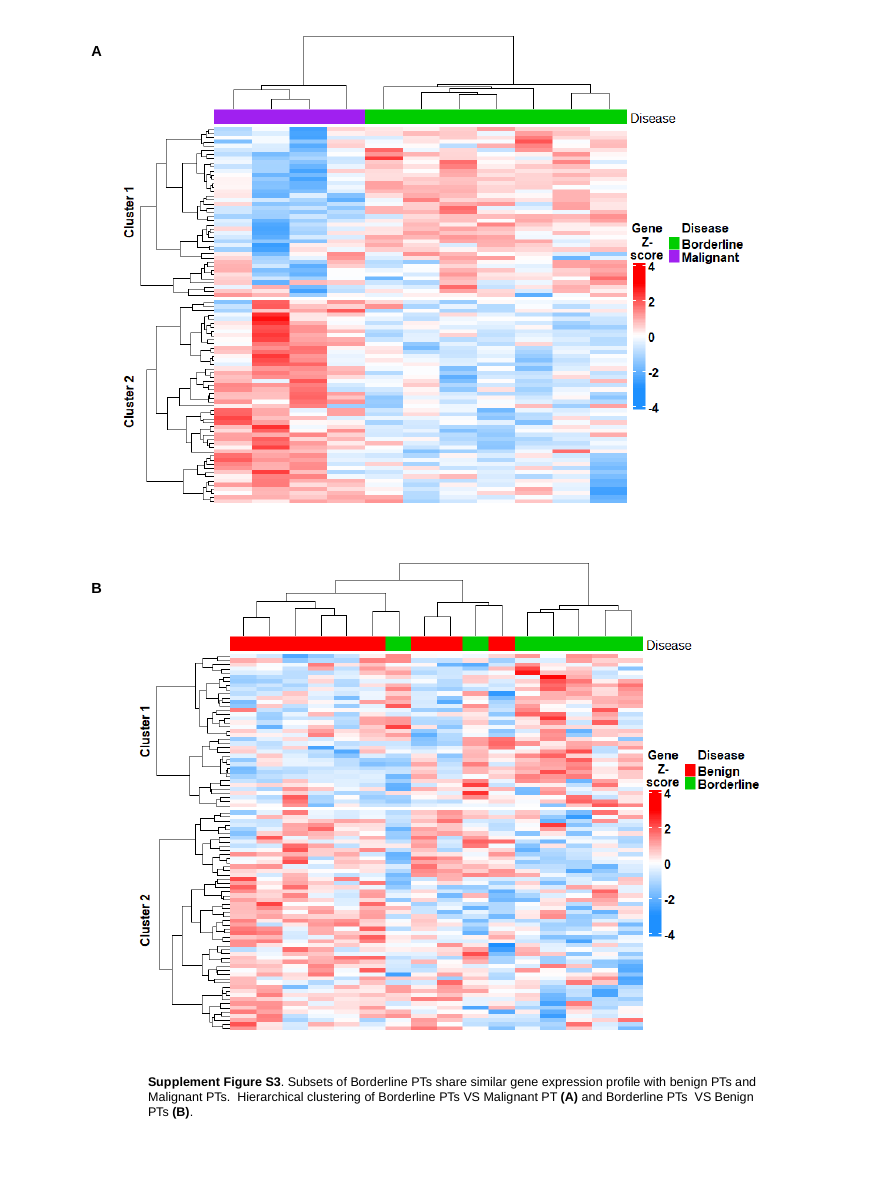

A
B
Supplement Figure S3. Subsets of Borderline PTs share similar gene expression profile with benign PTs and Malignant PTs. Hierarchical clustering of Borderline PTs VS Malignant PT (A) and Borderline PTs VS Benign PTs (B).
